# World Cancer Day 2021 - Perspectives in Pediatric and Adult Neuro-Oncology

**DOI:** 10.3389/fonc.2021.659800

**Published:** 2021-05-10

**Authors:** Erik P. Sulman, David D. Eisenstat

**Affiliations:** ^1^ Section of Neuro-oncology & Neurosurgical Oncology, Frontiers in Oncology and Frontiers in Neurology, Lausanne, Switzerland; ^2^ Department of Radiation Oncology, NYU Grossman School of Medicine, New York, NY, United States; ^3^ Brain and Spine Tumor Center, Laura and Isaac Perlmutter Cancer Center, New York, NY, United States; ^4^ NYU Langone Health, New York, NY, United States; ^5^ Children’s Cancer Centre, Royal Children’s Hospital, Parkville, VIC, Australia; ^6^ Murdoch Children’s Research Institute, Parkville, VIC, Australia; ^7^ Department of Paediatrics, University of Melbourne, Parkville, VIC, Australia

**Keywords:** neuro-oncology, neurosurgical oncology, oncology, neurology, pediatrics

## Abstract

Significant advances in our understanding of the molecular genetics of pediatric and adult brain tumors and the resulting rapid expansion of clinical molecular neuropathology have led to improvements in diagnostic accuracy and identified new targets for therapy. Moreover, there have been major improvements in all facets of clinical care, including imaging, surgery, radiation and supportive care. In selected cohorts of patients, targeted and immunotherapies have resulted in improved patient outcomes. Furthermore, adaptations to clinical trial design have facilitated our study of new agents and other therapeutic innovations. However, considerable work remains to be done towards extending survival for all patients with primary brain tumors, especially children and adults with diffuse midline gliomas harboring Histone H3 K27 mutations and adults with isocitrate dehydrogenase (IDH) wild-type, O^6^ guanine DNA-methyltransferase gene (*MGMT*) promoter unmethylated high grade gliomas. In addition to improvements in therapy and care, access to the advances in technology, such as particle radiation or biologic therapy, neuroimaging and molecular diagnostics in both developing and developed countries is needed to improve the outcome of patients with brain tumors.

## Introduction

If one could infer by attendance at major neuro-oncology conferences and the representation of pediatric and adult neuro-oncology at international oncology meetings, there has been an influx of new investigators, interest and significant advances in biomedical research pertaining to improving diagnosis, risk stratification, and treatment for children and adults with primary brain tumors. However, research progress has not yet had the anticipated impact on patient outcomes despite the promise. In the following article, we discuss several topics of current interest to the neuro-oncology community to reflect the directions the field is taking.

## Diagnostic and Prognostic Considerations

The 2016 update to the World Health Organization (WHO) Classification of Tumours of the Central Nervous System brought important refinements, including but not limited to molecular genetic subgroups of medulloblastoma and the introduction of diffuse midline glioma with Histone H3 K27 mutations (1). With the advent and subsequent implementation of platforms such as whole genome sequencing (2), single cell nucleic acid sequencing (3–7), nanostring technology (8, 9) and DNA methylation (10–12) profiling, some diagnostic categories have been replaced, such as the former primitive neuroepithelial tumor (PNET)grouping (13), whereas more common tumors such as low grade gliomas and glioblastoma (GBM) in pediatric and adult age groups are being split into subgroups specified by molecular and genetic considerations (14–20). The Glioma Longitudinal Analysis Consortium (**GLASS**) was established to assess genomic, epigenomic and other molecular changes such as tumor mutational burden and mutational signatures that occur over time from initial diagnosis to tumor progression/recurrence, including in response to chemotherapy and radiation (19, 21). Although driver mutations were retained at recurrence, prior therapies such as alkylating agents contributed to acquired mutations, including a hypermutator phenotype. Furthermore, selection of subclones with disease progression portended a worse prognosis (19). Other consortia, including the Consortium to Inform Molecular and Practical Approaches to CNS tumor Taxonomy (**cIMPACT-NOW**) (22, 23) have been organized to make further refinements that will be incorporated into the next edition of the WHO Classification.

Going forward, the task will be to prospectively study these subgroups in well designed clinical trials limited by smaller numbers of patients with these specific diagnoses. Significant pre-clinical and basic research is needed to identify actionable therapeutic targets within these subgroups. Furthermore, once appropriate therapies are identified, successful clinical trial accrual will likely require international collaboration given the limited patient numbers. However, many of these advanced molecular diagnostic technologies are not accessible in the developing world limiting the ability to both include these regions in trials and appropriately apply new treatments to the patients living there. Efforts to democratize molecular pathology using more widely available assays may be necessary, even at the cost of precision.

Other important advances include **liquid biopsy** for both initial diagnosis and at the time of progression/recurrence, such as for diffuse midline gliomas and to follow responses to therapy (24, 25). This is an important concept given the potential morbidity of repeated brain biopsy and the limitations of conventional magnetic resonance imaging (MRI). Challenges regarding the choice of cerebrospinal fluid (CSF), plasma, or serum, the technological platforms to utilize and which specific components (cell free DNA, RNA, microRNA, other noncoding RNAs, exosomes, tumor-educated platelets, etc.) remain as very active areas of investigation (26–28).

**Repeat biopsy or tumor resection** can be beneficial to the patient, including reduction of residual disease, assessment of acquired mutational profile and/or identification of new mutations (29). Timing of reoperation can influence the survival benefit and this should be factored into both retrospective and prospective studies (30). Reoperation may provide time to offer salvage therapies, including stereotactic radiosurgery, and assess their efficacy. However, the extent of re-resection is often limited by patient choice, the neuroanatomic location of the tumor and other considerations, such as risks of (further) neurological impairment, venous thromboembolism and/or other complications. Moreover, repeat biopsy may not provide sufficient tissue for full molecular genetic studies. Yet, this new data may inform the selection of available targeted therapies or enable the application of local therapies, such as oncolytic viruses, at the time of reoperation. The availability of additional genomic and epigenomic data includes the mutational signature associated with temozolomide and determination of tumor mutational burden (TMB). An increased TMB is one factor that may render the patient suitable for therapy with immune checkpoint inhibitors, discussed later in this Perspectives article. Furthermore, reoperation can facilitate eligibility to phase I/II clinical trials of novel targeted therapies or assessment of drug delivery and target inhibition in phase 0 or “window of opportunity” clinical trials (31).

**Bioinformatic** analyses of databases such as the Cancer Genome Atlas (TCGA) and the Chinese Glioma Genome Atlas (CGGA) have yielded numerous studies identifying novel prognostic and/or predictive biomarkers. However, many of these studies lack functional or clinical validation studies or have yet to be studied prospectively in clinical trials. Indeed, many of the molecular subgroups identified from these datasets reveal distinct biologies but are often defined by molecular techniques, such as whole transcriptome profiling, that are not readily applicable to the clinical setting.

## Neuroimaging and Neurosurgery

The use of chemoradiation and subsequently bevacizumab for adult GBM underscored the importance of identifying pseudoprogression and pseudoresponse, respectively. The Response Assessment in Neuro-Oncology (**RANO) criteria** (32, 33) and more recently **iRANO** (immunotherapy) (34) and **RAPNO** (pediatric) (35–37) working groups have standardized response assessments by neuroradiologists and other clinicians in the settings of both clinical trials and in the neuro-oncology clinic. These assessments have been particularly helpful in clinical trial design, but have less utility for the individual patient as the criteria often involve retrospective assignment of progression which is useful in determining the status of a clinical trial endpoint, but often too late to impact individual patient treatment.

The International Neuroimaging Data-sharing Initiative and others aim to streamline processing of MRI and other neuroimaging data across institutions following standard operating procedures for multi-institutional data sharing. These efforts are providing both neuroscientists and clinicians from less well-developed countries with access to advanced neuroimaging bioinformatics infrastructure, which can assist with diagnosis and assessment of responses to therapy (38–40). Collaborating approaches to develop segmentation algorithms (e.g. identifying areas of tumor or normal structures), such as the Federated Tumor Segmentation (FeTS) initiative (41), permit pooling of de-identified images and processing analysis tools to vastly improve upon what is possible from a single institution.

Furthermore, **radiomics** and the application of machine learning/artificial intelligence to diagnostic MRI scans has the potential to identify early tumor recurrence/progression, distinguish pseudoprogression from progression (42, 43) as well as to identify imaging signatures that are relatively specific to molecular subgroups of the more common diagnoses in adults (GBM, oligodendroglial tumors, low grade gliomas) (44, 45) and children (low grade gliomas, medulloblastoma, ependymoma, diffuse midline gliomas) (46, 47). While several techniques have been described, none have achieved widespread clinical acceptance for routine use. There remains a significant opportunity for those in the radiomics field to combine efforts and define standard, validated approaches to primary brain tumor imaging that can accurately predict tumor diagnosis as well as tumor progression. Once such radiomic collaboration to develop biomarkers of response is the Radiomics Signatures for Precision Diagnostics (ReSPOND) consortium (48) which, like the FeTS initiative, combines multiple institutional datasets to a much larger pool of data of over 3300 patients. Nevertheless, until these radiomic biomarkers achieve widespread clinical utilization, we are reliant on RANO criteria along with subjective clinical assessments.

**Intraoperative MRI** has the potential to increase the extent of resection and improve the delivery of local therapy, particularly when combined with direct intraoperative visualization techniques such as 5-aminolevulinic acid (5-ALA) **fluorescence guided surgery** (49, 50). Intraoperative stimulated Raman histology provides a real-time histologic analysis of tissue in under 60 seconds and can help direct the neurosurgeon, for example, to pursue additional biopsies or continue a more aggressive resection for a high-grade glioma (51, 52). **Focused ultrasound** can focally disrupt the blood brain barrier and also improve the provision of local therapies mediated by microbubbles (53, 54). Development of improved radiotracers for detection and/or therapy (theragnostic) of hypoxic, metabolic or specific molecular signatures by combined **PET-CT and PET-MR** systems is a very active area of preclinical and clinical study for intra-axial and extra-axial tumors of the central nervous system. For example, ongoing studies of ^177^Lu-DOTATATE in meningioma have the potential to change the course of this disease at recurrence (55, 56) (NCT03971461).

## Radiation Oncology

**Proton Beam Therapy (PBT)**, where available, has become the standard of care for some pediatric brain tumors, especially with the demonstration of improved outcomes with respect to hearing loss, neuroendocrinology and especially neurocognition (57–61). Craniospinal irradiation delivered *via* PBT has the advantages of relative sparing of the esophagus, bladder and bowel. However, although countries including the United Kingdom, Australia and Canada are planning to develop PBT in one or more sites, many developed countries currently lack dedicated proton therapy centers, so children and adults often have to travel very long distances to access this therapy (62). Furthermore, the place for PBT in adults, apart from generally accepted indications such as for chordomas, remains to be determined (63). There has been an observed trend to use PBT for low-grade and high-grade gliomas, the majority of which infiltrate into the surrounding brain parenchyma. Further study is warranted. Other forms of particle beam therapy, such as carbon ion therapy, are being evaluated in several countries for patients with meningiomas and gliomas and may have certain advantages over PBT, such as lower oxygen dependence (64).

Linear accelerators (LINAC) combined with onboard magnetic resonance imaging (MR/LINAC) units are increasing the precision of various radiation therapy modalities with the potential to reduce long-term sequelae. Moreover, these instruments allow for daily adaptation of treatments due to changes to tumor or normal anatomy or based on functional imaging data.

In the clinic, there has been a rising lower age limit to offer radiation to children and young adults with a brain tumor, respectively. Deferring or obviating the need for cranial irradiation in infants (less than 3 years) and young children (less than 10 years) is a very important consideration given the demonstrated impact of radiation on brain growth, development and cognition which continues through adolescence to young adulthood. However, it may be difficult to salvage patients with recurrent/progressive disease with radiation when it is not included in upfront therapies along with surgery and chemotherapy. Whenever possible, clinical trials accompanied by comprehensive neuropsychological and neurocognitive assessments are required when assessing the impact of reduced, delayed or omitted radiotherapy (65). For some patient populations, such as those with brain metastases, where therapeutic interventions often have limited impact on the patient’s survival but serve an important palliative role, the use of functional, neurocognitive endpoints takes on a greater significance (66). In trials of glioma patients where intermediate endpoints of progression based are of limited benefit, neurocognitive changes may serve as an early indicator of patient survival (67).

Non-ionizing radiation, such as **tumor treating fields** (TTFields), has shown a survival benefit for patients with newly diagnosed GBM (68) and to be equivalent to salvage chemotherapy for patients with recurrent GBM (69). Ongoing trials to combine TTFields with standard and novel therapies are being conducted in both adult and pediatric patients with brain tumors. Despite these results, ongoing concerns raised by some in neuro-oncology has limited its widespread adoption (70). However, recent positive clinical trials in other disease sites only highlight the role of TTFields in the oncologic armamentarium (71).

## Chemotherapy, Targeted and Epigenetic Therapies

The standard of care for newly diagnosed adults with glioblastoma, especially those with MGMT promoter methylated tumors, remains chemoradiation with temozolomide followed by 6 to 12 cycles of adjuvant temozolomide (72). However, the neuro-oncology community is eagerly awaiting a significant advance, especially for those with IDH wild-type MGMT promoter unmethylated tumors. A recent meta-analysis assessed the prognostic value of various MGMT promoter methylation tests for predicting overall survival in temozolomide treated GBM patients. Although both pyrosequencing and methylation specific polymerase chain reaction were superior to immunohistochemistry, determination of ideal thresholds and which specific CpG sites to assess remain undetermined (73).

Furthermore, there is no consensus with respect to the sequence and selection of chemotherapy and/or targeted therapies for recurrent GBM. However, the recent introduction of **IDH inhibitors** in advanced gliomas has demonstrated the importance of identifying molecular subgroups that can benefit from targeted therapies (74). The identification of less common GBM molecular subgroups with fusions involving FGFR or the TRK family of neurotrophin receptors has been another promising advance leading to ongoing clinical trials using fibroblastic growth factor receptor (**FGFR) or tropomyosin receptor kinase (TRK) inhibitors**, respectively (75–77). Similarly, the use of v-Raf murine sarcoma viral oncogene homolog B (**BRAF) inhibitors** for tumors harboring BRAF V600E mutations, including pediatric low grade gliomas, gangliogliomas, pleiomorphic xanthoastrocytomas and Langerhans Cell Histiocytosis, has extended survival for many of these patients (78). A novel approach targeting protein arginine methyltransferase 5 (PRMT5), including a brain-penetrant **PRMT5 inhibitor**, has shown promise in preclinical studies wherein a specific splicing signature in GBM may predict responses to this drug class *in vitro* and *in vivo* (79).

The demonstration that pilocytic astrocytomas are driven by MAPK signaling has resulted in the implementation of **BRAF and/or MEK inhibitors** at the time of initial diagnosis or at progression (78). However, similar to the treatment of recurrent GBM in adults, the timing, sequence and/or duration of the use of these targeted therapies in children requires further study in carefully designed clinical trials, including separate cohorts for patients with neurofibromatosis (NF) type 1. The effect of long-term inhibition of MAPK signaling on normal growth and development of the child remains undetermined. Furthermore, there still remains a place for single agent or combination chemotherapy for these relatively common pediatric brain tumors.

Advances in our understanding of the molecular genetics of diffuse midline gliomas and high-grade gliomas in children have identified the coopting of neurodevelopmental pathways by these tumors and underscore the importance of harnessing **epigenetic-based therapies**, including but not limited to selected HDAC, bromodomain and other inhibitors (80–82). Posterior fossa type A (PFA) ependymomas (83, 84) also demonstrate loss of Histone H3 K27 trimethylation and may benefit from the implementation of these treatments. Challenges are considerable, including tumor specificity, and international cooperative groups are focused on early phase clinical trials to identify promising agents to advance to larger patient cohorts.

## Clinical Trial Design

As former diagnostic categories are parsed into subgroups based upon molecular genetic and other diagnostic considerations, the field of neuro-oncology continues to explore other types of clinical trial design. These include **basket trials** where several diagnostic entities sharing the same mutational profile or target are grouped. **Umbrella trials** or **master protocols** allow larger groups of patients, for example adult GBM, to be enrolled in concurrent and/or sequential smaller phase II trials as part of one very large study that can more efficiently assess the efficacy of novel, often targeted therapies, either at diagnosis or at the time of tumor progression. Adaptive, Bayesian and other innovative clinical trial designs that optimize patient eligibility or use data from prior clinical trials are essential to rapidly translate progress from the basic laboratory to the clinic to improve patient outcomes (85–87). The ongoing Adaptive Global Innovative Learning Environment for Glioblastoma (GBM AGILE) trial combines adaptive trial design with a registration expansion cohort for rapid evaluation of candidate therapeutics and regulatory approval while minimizing the required patient sample size (88). A unique feature of GBM AGILE is the direct incorporation of molecular classification (namely MGMT promoter methylation status) into the trial and the potential for incorporation of treatment-specific predictive molecular biomarkers. This type of adaptive trial is a model which is applicable across neuro-oncology.

## Immuno-Oncology

It has been a very exciting time for innovative approaches using several types of therapy that harness the immune system, either alone, in combination or added to standard therapies using chemotherapy or radiation therapy (89, 90). These approaches include **tumor vaccines** (91), **oncolytic viruses** (92–96), **immune checkpoint inhibitors** (97–99) and chimeric antigen receptor (**CAR) T-cells** (100–103). Improved clinical outcomes using immune checkpoint inhibitors in patients with biallelic mismatch repair deficiency and high tumor mutation burdens (TMB) have been reported (104). However, many pediatric and some adult brain tumors have low TMB and are highly immunosuppressive. Recent negative reports of phase III trials of immune checkpoint inhibitors in GBM highlights this challenge (105, 106). Moreover, the use of immunotherapies is complicated by the potential for intracranial inflammation which may result in significant morbidity or long-term complications. Treatment of inflammation using standard corticosteroid therapy can further compound the tumor immunosuppression and negate any benefit from immunotherapy. Other factors under active study include assessment of the immune tumor microenvironment and how modulating the tumor microenvironment may improve the efficacy of these immunotherapies. Moreover, the influence of the variably intact blood brain barrier and the unintended adverse consequences of immunotherapy, such as brain edema, aseptic meningitis, encephalitis, or peripheral neuropathies are also important considerations as this very promising area of therapy is further developed.

## Awareness, Equity, Diversity and Inclusivity

In both developed and developing countries there are initiatives to raise public awareness of brain tumors, including the HeadSmart program in the United Kingdom (107). Access to emerging diagnostic (genomic platforms, DNA methylation profiling, advanced imaging) and therapeutic options (targeted and immunotherapies, PBT) remains limited to some developed countries or specific tertiary/quaternary pediatric or comprehensive cancer centers leading the vanguard in neuro-oncology (108). Moreover, it will be challenging for health care systems or third-party insurers in many countries to ensure equitable access to these recent and emerging clinical advances.

## Data Availability Statement

The original contributions presented in the study are included in the article/supplementary material. Further inquiries can be directed to the corresponding authors.

## Author Contributions

This submission is a Perspective article for World Cancer Day 2021 submitted by DE and ES who equally contributed to the submission. All authors contributed to the article and approved the submitted version.

## Conflict of Interest

The authors declare that the research was conducted in the absence of any commercial or financial relationships that could be construed as a potential conflict of interest.
